# Comparative effectiveness of 9 ovulation-induction therapies in patients with clomiphene citrate-resistant polycystic ovary syndrome: a network meta-analysis

**DOI:** 10.1038/s41598-017-03803-9

**Published:** 2017-06-19

**Authors:** Yiping Yu, Lanlan Fang, Ruizhe Zhang, Jingyan He, Yujing Xiong, Xiaoyi Guo, Qingyun Du, Yan Huang, Yingpu Sun

**Affiliations:** Department of Reproductive Medical Center, First Affiliated Hospital of Zhengzhou University, Zhengzhou, China

## Abstract

The comparative efficacies of ovulation-induction treatments in patients with clomiphene citrate-resistant (CCR) polycystic ovary syndrome (PCOS) are not well known. Therefore, we conducted a network meta-analysis to rank the reproductive efficacies of these treatments. We ultimately included 26 randomized clinical trials with 2722 participants and 9 types of therapies: clomiphene citrate (CC), metformin, letrozole, follicle stimulating hormone (FSH), human menopausal gonadotropin (hMG), unilateral laparoscopic ovarian drilling (ULOD), bilateral laparoscopic ovarian drilling (BLOD), the combination of metformin with letrozole (metformin+letrozole), and the combination of metformin with CC (metformin+CC). The network meta-analysis demonstrates that hMG therapy result in higher pregnancy rates than BLOD, ULOD and CC therapies. Pregnancy, live birth and ovulation rates are significantly higher in metformin+letrozole and FSH groups than CC group. The abortion rate in the metformin+letrozole group is significantly lower than that in the metformin+CC group. Ranking probabilities show that, apart from gonadotropin (FSH and hMG), metformin+letrozole is also potentially more effective in improving reproductive outcomes than other therapies. In conclusion, owing to the low quality of evidence and the wide confidence intervals, no recommendation could be made for the treatment of ovulation-induction in patients with CCR PCOS.

## Introduction

A large portion of polycystic ovary syndrome (PCOS) patients with clomiphene citrate-resistant (CCR) directly undergo *in vitro* fertilization (IVF) to get higher cumulative pregnancy rates^1^. However, PCOS patients are more likely to develop ovarian hyperstimulation syndrome (OHSS), which is a life-threatening complication. Thus, more efficacious ovulation therapies, especially mono-ovulation should be developed and verified in CCR-PCOS patients before referral to IVF to minimize the costs and the occurrence of OHSS and multiple pregnancies.

Several effective therapies have been introduced for the treatment of ovulation-induction in CCR-PCOS. First, gonadotropin, including follicle stimulating hormone (FSH) and human menopausal gonadotropin (hMG), have been regarded as the second choice in patients who are insensitive to CC^2^. However, it is counter-intuitive for clinicians to choose FSH or hMG because of the high occurrence of OHSS and multiple pregnancies associated with their use. Second, letrozole works by specifically and reversibly blocking the synthesis of oestrogen and inducing mono-ovulation^3^. However, the efficacy of letrozole therapy in CCR-PCOS patients remains unclear. Third, metformin may reduce insulin resistance and hyperandrogenism and improve metabolic conditions and reproductive outcomes^4^. However, the extent to which metformin therapy improves reproductive outcomes needs further investigation. Fourth, laparoscopic ovarian drilling (LOD) may improve hormone conditions by decreasing the concentrations of androgens and luteinizing hormone (LH) and increasing FSH concentrations in serum. However, the comparative efficacies of unilateral laparoscopic ovarian drilling (ULOD) and bilateral laparoscopic ovarian drilling (BLOD) are under fierce debate. Finally, combination therapies have been introduced and are widely used to induce ovulation in CCR-PCOS patients, such as the combination of metformin with CC (metformin+CC) and metformin with letrozole (metformin+letrozole). However, the comparative efficacies of these treatments also remain unclear. Herein, we took CC as the control to estimate the comparative efficacies of other treatments for women with CCR-PCOS.

Numerous pairwise meta-analyses have tried to identify the efficacies of the above-mentioned therapies in CCR-PCOS patients^5–9^. However, traditional meta-analyses are not based on the full range of all widely used therapies and give limited suggestions to choose the most efficacious therapy for mono-ovulation in CCR-PCOS patients. Here, we employed the method of multiple-treatment meta-analysis, also known as network meta-analysis, to integrate data from direct and indirect comparisons and rank treatment efficacies^10–12^. We aimed to provide clinically useful assessments of treatments that can be used to guide treatment decisions for CCR-PCOS patients.

## Results

### Overview of the Literature Search and Study Characteristics

**Figure 1:**
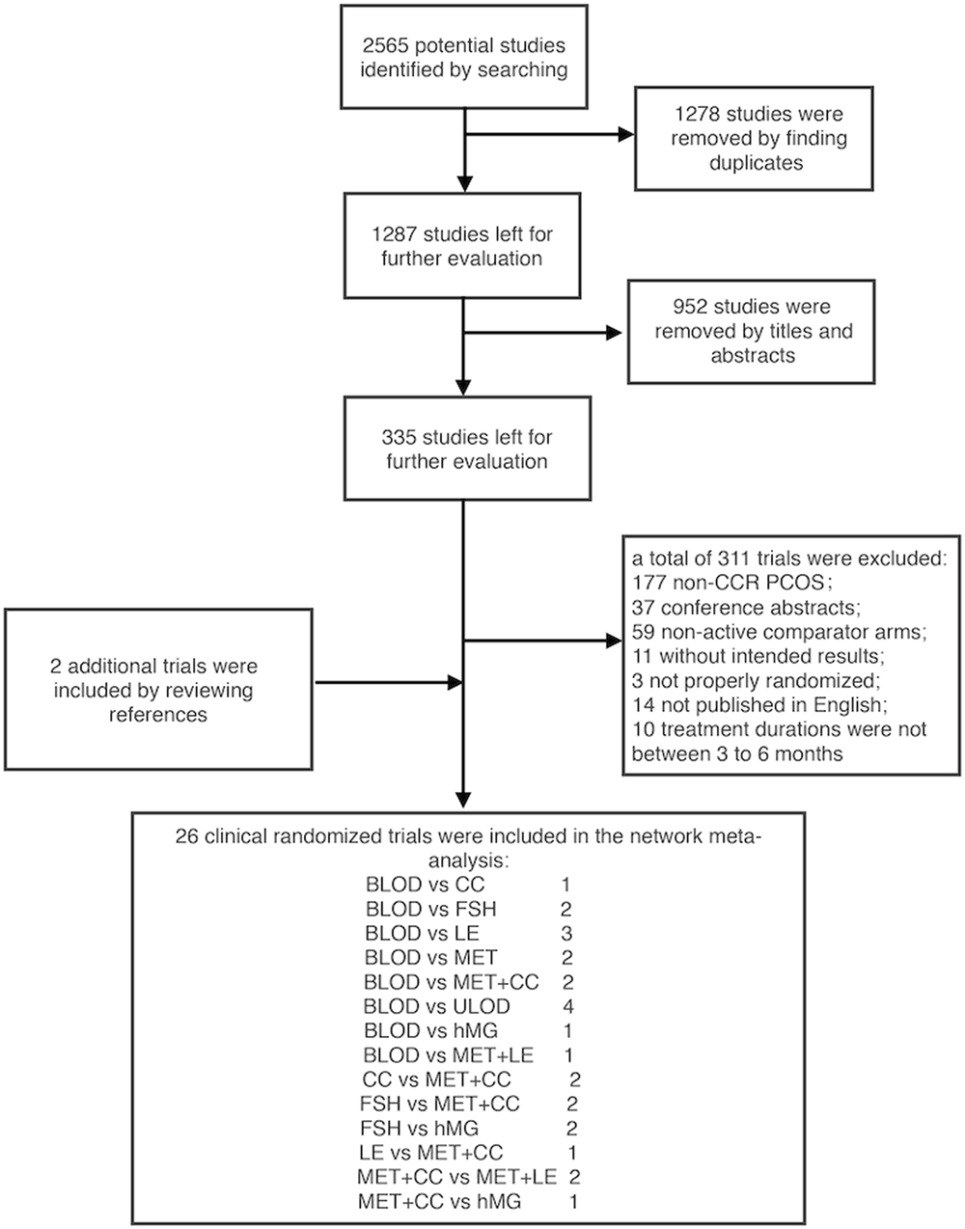
Flow diagram of the trial screening process. CC: clomiphene citrate, MET: metformin, LE: letrozole, FSH: follicle-stimulating hormone, hMG: human menopausal gonadotropin, MET+CC: metformin combined with clomiphene citrate, MET+LE: metformin combined with letrozole, ULOD: unilateral laparoscopic ovarian drilling and BLOD: bilateral laparoscopic ovarian drilling.

**Figure 2:**
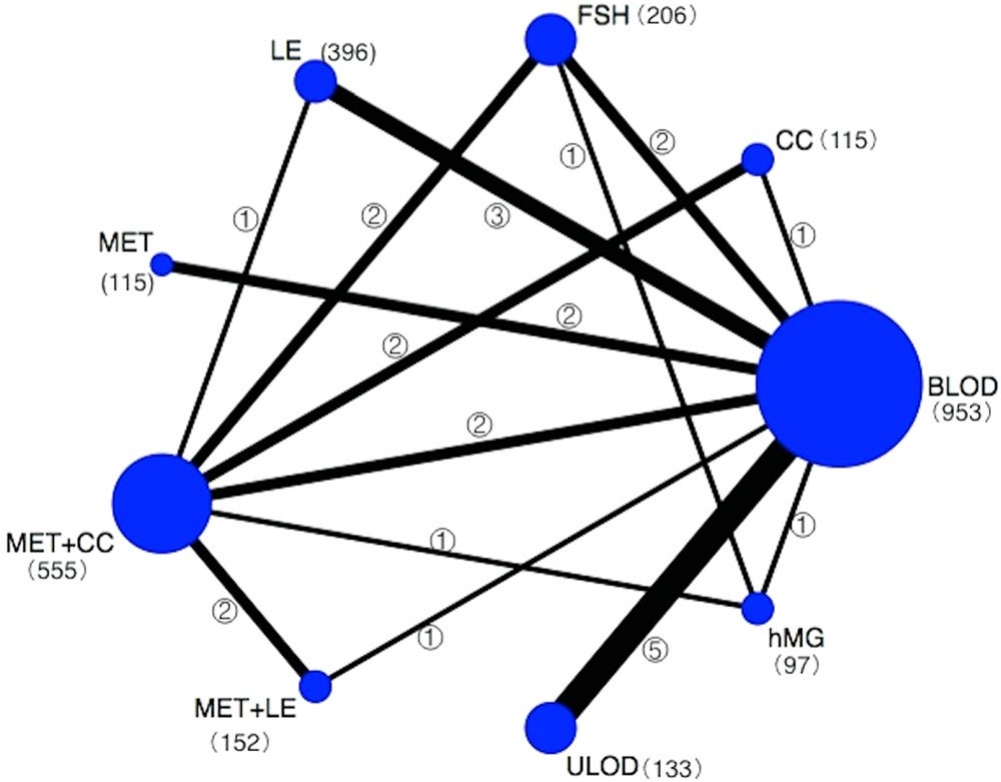
Network plot of the comparisons included in the analysis. CC: clomiphene citrate, MET: metformin, LE: letrozole, FSH: follicle-stimulating hormone, hMG: human menopausal gonadotropin, MET+CC: metformin combined with clomiphene citrate, MET+LE: metformin combined with letrozole, ULOD: unilateral laparoscopic ovarian drilling and BLOD: bilateral laparoscopic ovarian drilling. Numbers next to the treatment indicate participants undergoing a specific therapy; numbers on connecting lines between two comparisons indicate the number of direct comparisons.

A total of 2565 citations were retrieved based on electronic searches, and 2 additional studies were retrieved after checking the references of relevant reviews and guidelines. All citations were imported into EndNote X7. The screening process is shown in Fig. 1. Ultimately, 26 clinical trials with a total of 2722 participants were included in this multi-treatment meta-analysis; these trials explored the comparative efficacies of 9 therapies (CC, letrozole, metformin, metformin+letrozole, metformin+CC, ULOD, BLOD, FSH and hMG) for inducing mono-ovulation in CCR-PCOS patients^13–38^. The description of included trials is presented in Supplementary Table S1. A network plot was drawn to visually display the number of studies involved in each direct comparison and the total number of participants that received each treatment (Fig. 2).

### Assessment of Evidence Quality

**Figure 3:**
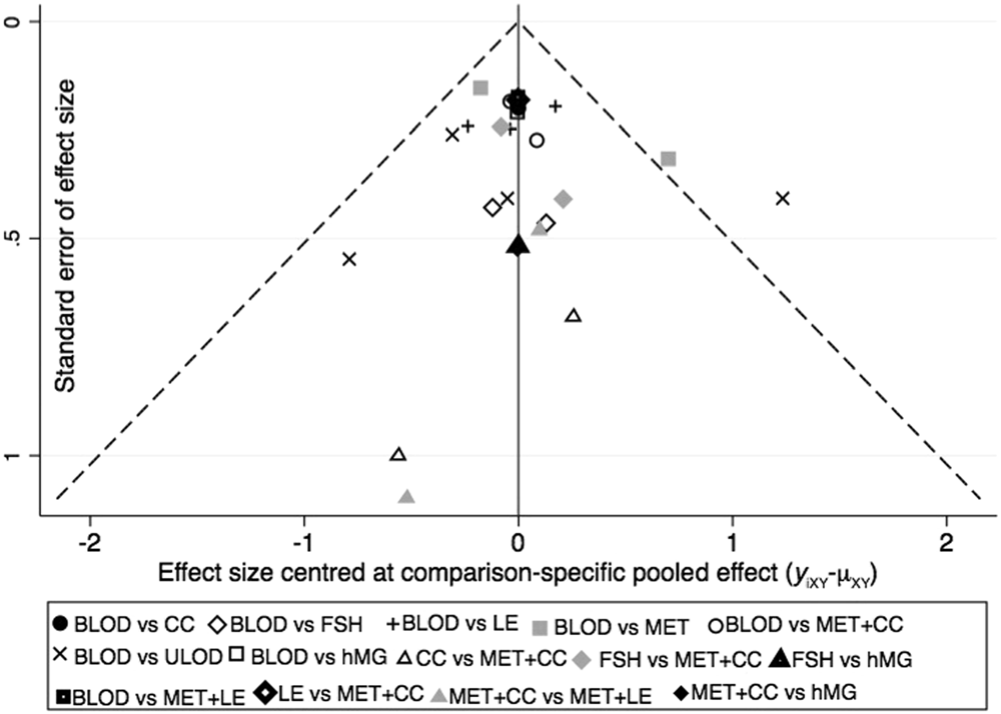
Funnel plot on pregnancy outcomes. CC: clomiphene citrate, MET: metformin, LE: letrozole, FSH: follicle-stimulating hormone, hMG: human menopausal gonadotropin, MET+CC: metformin combined with clomiphene citrate, MET+LE: metformin combined with letrozole, ULOD: unilateral laparoscopic ovarian drilling and BLOD: bilateral laparoscopic ovarian drilling.

Most of the trials reported their methods for randomization and allocation concealment. Our judgement about each item relating to a risk bias for each included trial is described in Supplementary Figure S1. The main biases were caused by a lack of blinding of participants. The funnel plot was visually unsymmetrical, which indicated possible publication bias (Fig. 3). The exclusion of conference abstracts and inclusion of only English-language articles in this analysis might account for the observed publication bias.

The overall quality of this network meta-analysis was estimated according to the GRADE four-step approach. For each comparison, the direct, indirect and network estimates are presented in Table 1. Among indirect comparisons, significant inconsistencies were detected in the closed loop of BLOD-CC-metformin+CC (Supplementary Figure S2). Meanwhile, significant heterogeneities were detected in the direct comparisons of BLOD versus metformin and BLOD versus ULOD (Table 2). The detailed reasons for down rating for direct, indirect and network estimates are presented in Supplementary Table S2. Of all the 36 comparisons, 22 (61.1%) were rated at low quality and 14 (38.9%) were rated at very low quality. The main reasons for downgrading were the small sample size (imprecision) and lack of blinding of participants (limitations in design). Nevertheless, it would be unethical to utilize some form of blinding method while comparing BLOD with drug therapies (12 trials).

**Table 1:** Ratings of direct, indirect and network meta-analysis according to the GRADE.

Comparisons	Quality of direct evidence	Quality of indirect evidence	Quality of NMA
BLOD vs. CC	⊕⊕⊙⊙ low^1,4^	⊕⊙⊙⊙ very low^1,2,4^	low
BLOD vs. FSH	⊕⊙⊙⊙ very low^1,44^	⊕⊕⊙⊙ low^1,4^	low
BLOD vs. LE	⊕⊕⊙⊙ low^1,4^	⊕⊕⊙⊙ low^1,4^	low
BLOD vs. MET	⊕⊙⊙⊙ very low^1,2,4,5^	/	very low
BLOD vs. MET + CC	⊕⊕⊙⊙ low^1,4^	⊕⊕⊙⊙ low^1,4^	low
BLOD vs. ULOD	⊕⊙⊙⊙ very low^1,2,4,5^	/	very low
BLOD vs. hMG	⊕⊕⊙⊙ low^1,4^	⊕⊕⊙⊙ low^1,4^	low
BLOD vs. MET + LE	⊕⊕⊙⊙ low^1,4^	⊕⊕⊙⊙ low^1,4^	low
CC vs. FSH	/	⊕⊕⊙⊙ low^1,4^	low
CC vs. LE	/	⊕⊕⊙⊙ low^1,4^	low
CC vs. MET	/	⊕⊕⊙⊙ low^1,4^	low
CC vs. MET + CC	⊕⊕⊙⊙ low^44^	⊕⊙⊙⊙ very low^1,2,4^	low
CC vs. ULOD	/	⊕⊙⊙⊙ very low^1,2,4,5^	very low
CC vs. hMG	/	⊕⊙⊙⊙ very low^1,44^	very low
CC vs. MET + LE	/	⊕⊙⊙⊙ very low^1,44^	very low
FSH vs. LE	/	⊕⊕⊙⊙ low^1,4^	low
FSH vs. MET	/	⊕⊕⊙⊙ low^1,4^	low
FSH vs. MET + CC	⊕⊕⊙⊙ low^1,4^	⊕⊕⊙⊙ low^1,4^	low
FSH vs. ULOD	/	⊕⊙⊙⊙ very low^1,4,5^	very low
FSH vs. hMG	⊕⊙⊙⊙ very low^1,44^	⊕⊕⊙⊙ low^1,4^	low
FSH vs. MET + LE	/	⊕⊕⊙⊙ low^1,4^	low
LE vs. MET	/	⊕⊕⊙⊙ low^1,4^	low
LE vs. MET + CC	⊕⊕⊙⊙ low^1,4^	⊕⊕⊙⊙ low^1,4^	low
LE vs. ULOD	/	⊕⊙⊙⊙ very low^1,4^	very low
LE vs. hMG	/	⊕⊕⊙⊙ low^1,4^	low
LE vs. MET + LE	/	⊕⊕⊙⊙ low^1,4^	low
MET vs. MET + CC	/	⊕⊙⊙⊙ very low^1,4^	very low
MET vs. ULOD	/	⊕⊙⊙⊙ very low^1,4^	very low
MET vs. hMG	/	⊕⊕⊙⊙ low^1,4^	very low
MET vs. MET + LE	/	⊕⊙⊙⊙ very low^1,2,4^	very low
MET + CC vs. ULOD	/	⊕⊙⊙⊙ very low^1,2,4^	very low
MET + CC vs. hMG	⊕⊙⊙⊙ very low^1,4,5^	⊕⊕⊙⊙ low^1,4^	low
MET + CC vs. MET + LE	⊕⊙⊙⊙ very low^1,44^	⊕⊕⊙⊙ low^1,4^	low
ULOD vs. hMG	/	⊕⊙⊙⊙ very low^1,2,4^	very low
ULOD vs. MET + LE	/	⊕⊙⊙⊙ very low^1,2,4^	very low
hMG vs. MET + LE	/	⊕⊕⊙⊙ low^1,4^	low

CC: clomiphene citrate, MET: metformin, LE: letrozole, FSH: follicle-stimulating hormone, hMG: human menopausal gonadotropin, MET+CC: metformin combined with clomiphene citrate, MET+LE: metformin combined with letrozole, ULOD: unilateral laparoscopic ovarian drilling, BLOD: bilateral laparoscopic ovarian drilling. ^1^limitations in design; ^11^serious limitations in design; ^2^inconsistency; ^3^indirectness; ^4^imprecision; ^44^serious limitations in imprecision; ^5^publication bias. All the indirect comparisons here are transportable, so ^3^indirec﻿tness is not a main reason for down-grading.

**Table 2:** Outcomes of direct comparisons of pregnancy rates, live birth rates, ovulation rates, abortion rates and multiple pregnancy rates.

Comparison	Pregnancy rate			Live birth rate			Abortion rate			Ovulation rate			Multiple pregnancy rate		
	Num. of trials	OR (95% CI)	I^2^(%)	Num. of trials	OR (95% CI)	I^2^(%)	Num. of trials	OR (95% CI)	I^2^(%)	Num. of trials	OR (95% CI)	I^2^(%)	Num. of trials	OR (95% CI)	I^2^(%)
**BLOD vs**															
CC	1	1.16(0.78–1.72), p = 0.46	0	1	1.15(0.73–1.80), p = 0.55	0	1	1.06(0.359–3.12), p = 0.92	0	0	—	—	1	0.10(0.01–1.76), p = 0.12	0
FSH	2	0.92(0.50–1.72), p = 0.80	0	1	0.72(0.20–2.57), p = 0.62	0	1	1.17(0.39–3.51), p = 0.78	0	0	—	—	1	0.20(0.01–3.46), p = 0.27	0
LE	3	1.19(0.92–1.54), p = 0.18	0	3	0.79(0.60–1.04), p = 0.10	17.2	3	1.62(0.70–3.78), p = 0.26	0	3	0.90 (0.75–1.07), p = 0.23	89.7	1	0.50(0.02–11.67), p = 0.67	0
MET	2	1.18(0.49–2.88), p = 0.71	84.7	2	1.05(0.35–3.13), p = 0.94	88	2	1.61(0.74–3.52), p = 0.23	0	2	1.21(0.78–1.88), p = 0.40	90.8	0	—	—
MET + CC	2	1.06(0.78–1.44), p = 0.70	0	1	1.08(0.62–1.89), p = 0.78	0	2	0.80(0.38–1.70), p = 0.56	0	2	0.91(0.71–1.17), p = 0.48	79.8	1	0.10(0.01–1.86), p = 0.12	0
ULOD	4	1.31(0.58–2.94), p = 0.51	77.4	2	1.17(0.65–2.11), p = 0.61	0	3	1.02(0.42–2.49), p = 0.96	0	4#	1.31(0.59–2.94), p = 0.51	77.4	0	—	—
hMG	1	**0.49(0.32**–0.73), p = 0.00*	0	0	—	—	1	1.99(0.60–4.87), p = 0.31	0	0	—	—	1	0.41(0.05–3.26), p = 0.40	0
MET + LE	1	0.97(0.69–1.37), p = 0.87	0	0	—	—	1	0.77(0.19–3.20), p = 0.72	0	0	—	—	0	—	—
**CC vs**															
MET + CC	2	**0.22(0.07**–0.65), p = 0.01*	0	1	0.20(0.03–1.52), p = 0.12	—	1	0.70(0.05–9.41), p = 0.79	0	1	**0.36(0.23**–**0.56), p = 0.00**	0	0	—	—
**FSH vs** –															
MET + CC	2	**1.87(1.23**–2.83), p = 0.00*	0	2	**2.00(1.23**–3.25), p = 0.01*	0	2	0.83(0.30–2.31), p = 0.73	0	1	**1.35(1.20**–1.53), p = 0.00*	0	0	1.69(0.38–7.51), p = 0.49	0
hMG	1	0.53(0.19–1.47), p = 0.22	0	1	0.86(0.18–4.01), p = 0.85	—	1	2.57(0.13–52.12), p = 0.54	0	—	—	—	1	0.29(0.01–5.79), p = 0.42	0
**LE vs**															
MET + CC	1	0.96(0.67–1.37), p = 0.82	0	1	0.88(0.25–3.07), p = 0.84	0	1	1.08(0.29–4.01), p = 0.91	0	1	0.93(0.83–1.04), p = 0.22	0	1	0.15(0.01–2.88), p = 0.21	0
**MET+CC vs**															
MET + LE	2	0.42(0.18–1.01), p = 0.05	0	1	**0.29(0.09**–0.95), p = 0.04*	0	2	**8.42(1.12**–63.52), p = 0.04*	0	1	**0.53(0.41**–0.69), p = 0.00*	0	0	—	—
hMG	1	0.71(0.26–2.00), p = 0.52	0	0	—	—	0	—		0	—	—	1	—	—

CC: clomiphene citrate, MET: metformin, LE: letrozole, FSH: follicle-stimulating hormone, hMG: human menopausal gonadotropin, MET+CC: metformin combined with clomiphene citrate, MET+LE: metformin combined with letrozole, ULOD: unilateral laparoscopic ovarian drilling, BLOD: bilateral laparoscopic ovarian drilling. CI: confidence interval; p<0.05* indicates significant difference. “—” indicates “not available”. #: ovulation rate per intention to treat.

In addition, all of our outcomes were objective indicators; therefore, biases caused by subjective estimations were limited. Meanwhile, the trials were all conducted in colleges or hospitals without the sponsorship of pharmaceutical companies, and the avoidance of sponsorship bias enhanced the validity of our network meta-analysis. All of the above factors were taken into consideration while estimating the applicability of the evidence. In general, the overall evidence supporting the comparative effectiveness of the included therapies is low; therefore, the results of network meta-analysis and ranking probabilities are likely to be associated with uncertainty.

### Results of Direct Comparisons

Overall, 14 direct comparisons and 22 indirect comparisons were found in the 26 trials. All 26 trials reported the number of patients who became pregnant using experimental therapies. Among all trials, 17 provided information on live birth rate per intention to treat (ITT) and compared all 9 treatments, 21 reported abortion rates per pregnancy and compared all 9 treatments, 11 reported ovulation rates per cycle and compared 7 treatments (data for ULOD and hMG could not be obtained), and 8 trials reported multiple pregnancy rates and compared 6 treatments (data for ULOD, metformin and metformin+letrozole could not be obtained).

**Figure 4:**
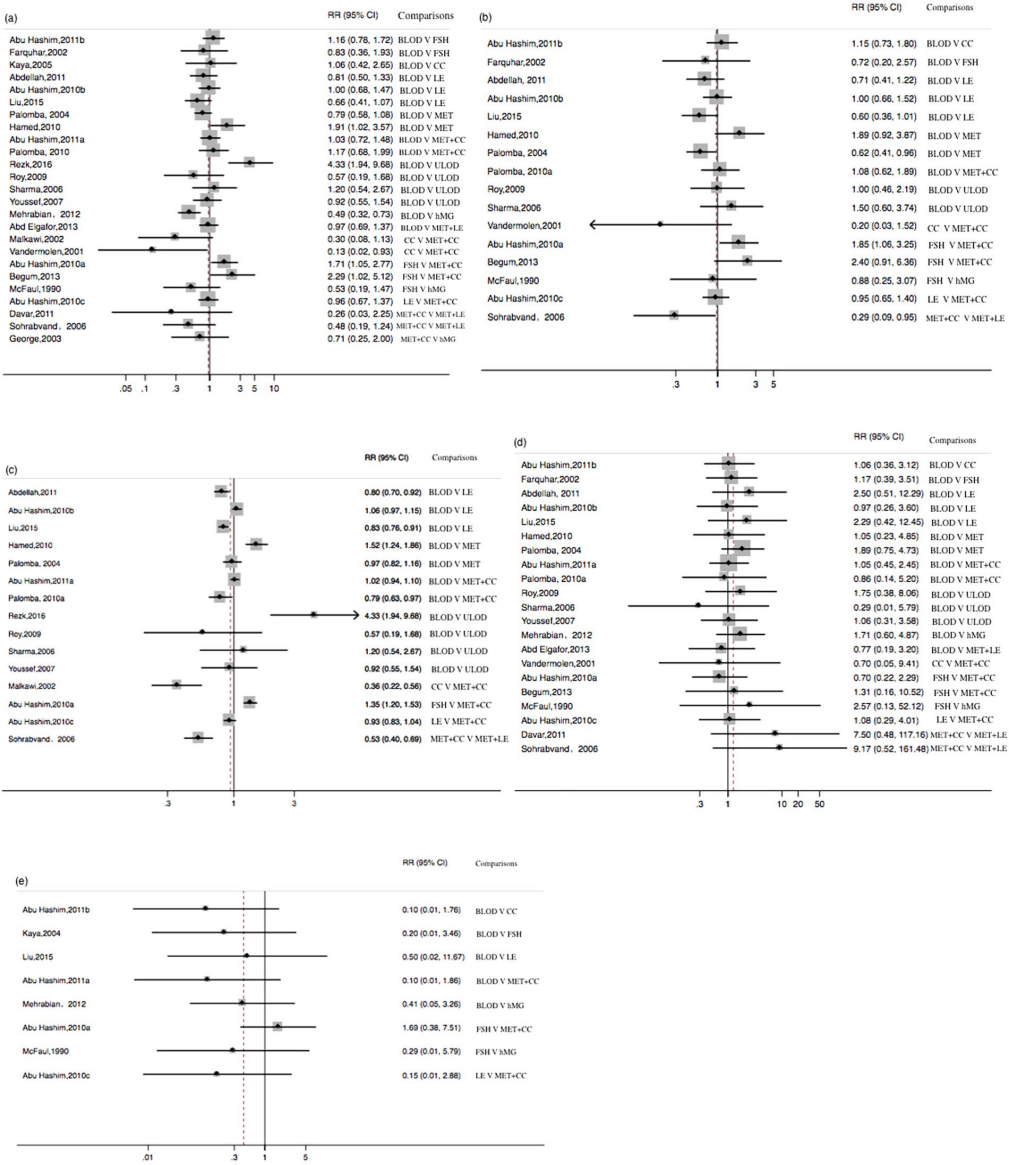
Forest plots showing effects of treatments for all direct comparisons. (**a**) pregnancy rate per ITT. (**b**) live birth rate per ITT. (**c**) ovulation rate per cycle, for BLOD verus ULOD: ovulation rate per ITT. (**d**) abortion rate per pregnancy. (**e**) multiple pregnancy rate per pregnancy. CC: clomiphene citrate, MET: metformin, LE: letrozole, FSH: follicle-stimulating hormone, hMG: human menopausal gonadotropin, MET+CC: metformin combined with clomiphene citrate, MET+LE: metformin combined with letrozole, ULOD: unilateral laparoscopic ovarian drilling and BLOD: bilateral laparoscopic ovarian drilling, CI: confidence interval.

We performed traditional pairwise meta-analyses by synthesizing trials that compared the same interventions using random effect or fixed effect models. The results of this analysis are shown in Table 2. The forest plots are shown in Fig. 4. Direct comparisons showed that metformin+CC therapy was significantly less efficacious than metformin+letrozole therapy in increasing live birth rates (0.29 (0.09–0.95), p = 0.04) and ovulation rates (0.53 (0.41–0.69), p = 0.00). Abortion rates (8.42 (1.12–63.52), p = 0.04) were significantly higher in metformin+CC therapy than metformin+letrozole therapy. In addition, metformin+letrozole therapy showed a greater efficacy in improving pregnancy rates than metformin+CC therapy (0.42 (0.18–1.01), p = 0.05). Ovulation rates (1.35 (1.20–1.53), p = 0.00), live birth rates (2.00 (1.23–3.25), p = 0.01) and pregnancy rates (1.87 (1.23–2.83), p = 0.00) were all significantly higher in the FSH group than in the metformin+CC group. Reported pregnancy rates were significantly lower in patients who underwent BLOD than in patients who underwent hMG therapy (0.49 (0.32–0.73), p = 0.00). Furthermore, ULOD was similar to BLOD with regard to pregnancy rates (1.31(0.58–2.94), p = 0.51), live birth rates (1.17 (0.65–2.11), p = 0.61), abortion rates (1.02 (0.42–2.49), p = 0.96) and ovulation rates (1.31(0.59–2.94), p = 0.51).

### Bayesian Network Meta-Analysis

**Figure 5:**
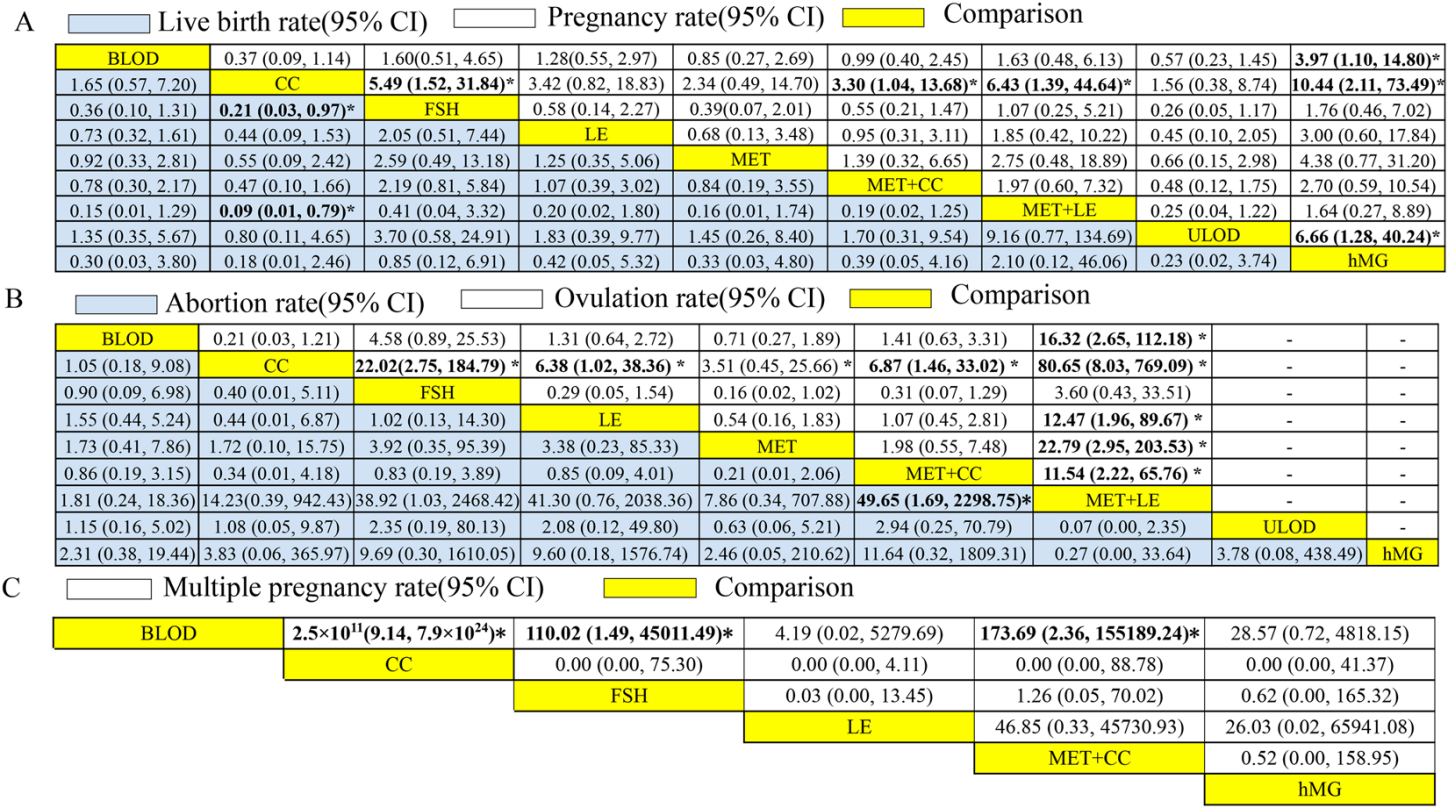
Results of network meta-analysis. Results are shown as OR (95%CI), representing column-defining treatment versus row-defining treatment. For pregnancy rates, live birth rates and ovulation rates, ORs higher than 1 favoured the column-defining treatment. For abortion rates and multiple pregnancy rates, ORs lower than 1 favoured the column-defining treatment. Significant results are shown in bold with “*” at the top right corner. CC: clomiphene citrate, MET: metformin, LE: letrozole, FSH: follicle-stimulating hormone, hMG: human menopausal gonadotropin, MET+CC: metformin combined with clomiphene citrate, MET+LE: metformin combined with letrozole, ULOD: unilateral laparoscopic ovarian drilling and BLOD: bilateral laparoscopic ovarian drilling. CI: confidence interval. “-” indicates unavailable.

We performed node-splitting analyses to assess the consistencies and inconsistencies in the network meta-analysis. Inconsistencies were identified between the direct and indirect comparisons for pregnancy rates per ITT. When an evident inconsistency was detected, an inconsistency model would be adopted, otherwise, a consistency model. After verifying the extracted data, many potential factors were identified as potentially being associated with the observed inconsistencies; the use of various dosages in different trials (Supplementary Table S1) and the small sample sizes were all identified as potential factors. The outcomes of the network analyses for pregnancy rates, live birth rates, ovulation rates per cycle, multiple pregnancy rates per pregnancy and abortion rates per pregnancy are shown in Fig. 5.

FSH and metformin+letrozole therapies were both identified as more efficacious than CC therapy in improving pregnancy rates and live birth rates. Furthermore, pregnancy rates in hMG groups were significantly higher than those in BLOD, ULOD and CC groups. Ovulation rates were significantly higher in metformin+letrozole groups than in BLOD, CC, letrozole, metformin and metformin+CC groups. The network meta-analysis of abortion rates indicated that abortion rates in metformin+letrozole groups were significantly lower than in metformin+CC groups. In terms of multiple pregnancy rates, patients who underwent BLOD were significantly less likely to conceive multiple babies than patients who underwent CC, FSH or metformin+CC therapies. A quantitative analysis of the occurrence of adverse events and OHSS was not performed due to the limited data extracted. In general, most adverse effects occurred during the administration of metformin and mainly included nausea and vomiting, which was tolerable to most participants; the occurrence of OHSS was mainly observed during gonadotropin therapies (see Supplementary Table S1). However, the results of network meta-analysis had very wide confidence interval (CI); it indicated insufficient power.

The ranking probabilities of the evaluated outcomes, including pregnancy rates per ITT, live birth rates per ITT, ovulation rates per cycle, abortion rates per pregnancy and multiple pregnancy rates per pregnancy, were all performed using ADDIS software version 1.16.7. The highest and second highest probabilities within each treatment rank are shown in Table 3. Based on the ranking probabilities, hMG and metformin+letrozole therapies had the highest probabilities of ranking first in the comparisons of pregnancy rates (0.71 and 0.17, respectively) and live birth rates (0.26 and 0.64, respectively) and the highest probabilities of ranking last in comparisons of abortion rates (0.27 and 0.42, respectively). Metformin+letrozole and FSH therapies had the highest probabilities of ranking first in comparisons of ovulation rates per cycle (0.89 and 0.1, respectively) (note: hMG therapy was excluded from this analysis because relevant data could not be obtained). In general, the three most efficacious therapies were hMG, FSH and metformin+letrozole with regard to reproductive outcomes. The three least efficacious therapies were CC, ULOD and BLOD. Additionally, the results of the network meta-analysis were in agreement with the results of pairwise comparisons.

**Table 3:** The first and second highest probabilities for each ranking over treatment.

Ranking	Pregnancy rate per intention to treat (probability)	Live birth rate per intention to treat (probability)	Ovulation rate per cycle	Abortion rate per pregnancy	multiple pregnancy rate
1	hMG(0.71) MET + LE(0.17)	MET + LE(0.64) hMG(0.26)	MET + LE(0.89) FSH(0.1)	MET + CC(0.32) ULOD(0.21)	CC(0.88) MET + CC(0.03)
2	FSH(0.35) MET + LE(0.30)	FSH(0.37) hMG(0.30)	FSH(0.82) MET + LE(0.09)	MET + CC(0.25) FSH(0.19)	MET + CC(0.29) FSH(0.28)
3	FSH(0.29) MET + LE(0.24)	FSH(0.41) hMG(0.16)	MET + CC(0.52) LE(0.34)	BLOD(0.22) MET + CC(0.17)	FSH(0.39) MET + CC(0.33)
4	LE(0.24) MET + CC(0.20)	MET + CC(0.27) LE(0.26)	LE(0.43) MET + CC(0.31)	BLOD(0.26) LE(0.14)	hMG(0.34) FSH(0.23)
5	BLOD(0.28) MET + CC(0.24)	LE(0.28) MET + CC(0.24)	BLOD(0.62) LE(0.13)	BLOD(0.21) LE(0.15)	LE(0.37) BLOD(0.33)
6	BLOD(0.35) MET + CC(0.22)	BLOD(0.28) MET + CC(0.17) LE(0.17)	MET(0.68) BLOD(0.15)	LE(0.18) MET (0.14)	BLOD(0.65) LE(0.32)
7	MET (0.26) MET + CC(0.18) ULOD(0.18)	BLOD(0.34) MET (0.17)	CC(0.90) MET(0.08)	hMG(0.19) MET(0.18)	—
8	ULOD(0.45) MET(0.20)	CC(0.27) ULOD(0.24)	—	hMG(0.25) MET + LE(0.20) MET(0.20)	—
9	CC(0.72) ULOD(0.2)	CC(0.47) ULOD(0.32)	—	MET + LE(0.42) hMG(0.27)	—

For pregnancy rate, live birth rate and ovulation rate, rank 1 was the best, and rank N was the worst. For abortion rate and multiple pregnancy rate, rank N was the best, and rank 1 was the worst. The higher the probability was, the more likely it ranked the position. CC: clomiphene citrate, MET: metformin, LE: letrozole, FSH: follicle-stimulating hormone, hMG: human menopausal gonadotropin, MET + CC: metformin combined with clomiphene citrate, MET + LE: metformin combined with letrozole, ULOD: unilateral laparoscopic ovarian drilling and BLOD: bilateral laparoscopic ovarian drilling.

### Sensitivity analyses

The ranking probabilities of this network meta-analysis might be unconvincing because the evidence was rated at very low to low quality according to GRADE. We therefore conducted sensitivity analyses to evaluate the stabilities of ranking probabilities by removing each of the trials. Although results of ranking probabilities were not completely stable, the comparative efficacies of included therapies could be demonstrated (Supplementary Table S4). In hMG, metformin+letrozole and FSH groups, the pregnancy rate and live birth rate remained higher and abortion rate remained lower than other groups. What was more, the ovulation rate remained steadily higher in metformin+letrozole and FSH groups. The result of sensitivity analysis for multiple pregnancies was shown to be unstable because of limited trials and participants included. Therefore, apart from hMG and FSH, metformin+letrozole showed potential efficacies in improving reproductive efficacies in patients with CCR-PCOS.

## Discussion

The present network meta-analysis provides evidence based on up-to-date clinical trials and allows for the comparisons of widely used but controversial clinical therapies. However, results of this network meta-analysis and ranking probabilities should be interpreted with caution, because the evidence was rated at very low to low quality. The main reasons for downgrading were serious imprecision and limitations in study design. In addition, the results of the network meta-analysis had very wide CIs, which indicated insufficient power. The results of sensitivity analyses of ranking probabilities were shown to be relatively stable with minor changes in pregnancy rates, live birth rates and ovulation rates. Although the evidence was still insufficient to make any conclusion, the potential efficacy of metformin+letrozole relative to other therapies was demonstrated for the first time.

Clinical consensus suggests that gonadotropin therapy (hMG or FSH), aromatase inhibitors (usually letrozole), LOD and adding metformin are common options for CCR patients. It is still challenging to induce mono-ovulation with gonadotropin therapies even under careful monitoring. In order to avoid multiple pregnancies and OHSS, many patients are asked to cancel cycles and refrain from sexual intercourse. Therefore, it is important to compare the comparative efficacies of above therapies to simplify clinical activities.

Cochrane systemic reviews and meta-analyses comparing different types of gonadotropin therapies indicated no significant difference between FSH and hMG in improving live birth rates in both CCR-PCOS and non-CCR PCOS^5, 39^. However, the above evidences were at low or very low quality. Another meta-analysis with low to moderate quality evidence demonstrated no differences in reproductive outcomes among metformin+CC, BLOD and letrozole^7^; gonadotropin therapy was more effective in improving ovulation rate and pregnancy rate than them^7^; it was similar to our results. Nevertheless, a Cochrane systemic review and meta-analysis conducted by Farquhar *et al*. comparing BLOD with ULOD, metformin+CC, gonadotropin therapy and aromatase inhibitors revealed that there was no evidence of differences in live birth rate between them^8^. But the multiple pregnancy rate was significantly lower in LOD groups^8^, which was consistent with the results of our ranking probabilities and another meta-analysis^40^. In a 2014 Cochrane review and a 2015 meta-analysis, it was demonstrated that, in non-CCR PCOS patients, the pregnancy rate and live birth rate were significantly higher with letrozole than with CC^41, 42^. However, the evidence was of low quality and findings should be interpreted with cautions especially in CCR-PCOS patients. In addition, there was either no difference or insufficient evidence for the comparisons of metformin with CC, placebo and other therapies in PCOS patients^43–45^.

Given the availability of RCTs that comparing different ovulation-induction treatments in CCR-PCOS patients, we undertook this network meta-analysis and were still unable to detect the most efficacious therapy. Our direct comparisons showed that metformin+CC could significantly improve pregnancy rate than CC (0.22(0.07–0.65), p = 0.01, 2 trials). Metformin+letrozole tend to improve pregnancy rate (0.42(0.18–1.01), p = 0.05, 2 trials) and live birth rate (0.29(0.09–0.95), p = 0.04, 1 trials) than metformin+CC. However, the findings should be strengthened by methodologically rigorous trials. The results of network meta-analysis were inconclusive because of the wide CIs. The evidence for the comparisons of abortion rate, ovulation rate, multiple pregnancy rate and adverse effects rate was insufficient; however, gastrointestinal adverse events occurred more frequently with metformin than others. In addition, OHSS mainly happened during the administration of gonadotropins therapy (Supplementary Table S1).

Sensitivity analyses were conducted to estimate the stability of ranking probabilities by removing each of the trials. The ranking probabilities of pregnancy rate, live birth rate and ovulation rate were relatively stable across sensitivity analyses with some minor changes. It was likely to be true that, apart from hMG and FSH, metformin+letrozole was potentially more effective in improving pregnancy rate, live birth rate and ovulation rate than other therapies.

No malformations were reported in offspring in included trials. However, safety is one of the most important factors to be considered during drug assessments. Novartis *et al*. previously questioned the use of letrozole therapy, because they claimed that they had observed an increased number of birth defects in patients treated with letrozole. However, the authors of many other canonical studies had voiced different opinions, demonstrating that a significantly increased incidence of congenital cardiac defects was observed in patients treated with CC^46, 47^. Taken together, the safety of CC and letrozole with regard to offspring health needs further studies to clarify this issue. The safety of metformin therapy was estimated as unclear by the US Food and Drug Administration (FDA). The meta-analysis conducted by Cassina *et al*. showed that metformin did not increase the risk of birth defects^48^.

Clinically, the assessment of efficacy during periods shorter than 3 cycles or longer than 6 cycles might vary widely. However, physicians usually initiate other therapies if certain drugs do not work well during the course of 3 to 6 cycles. In previous systemic reviews, treatments of different durations were combined, which limited the ability to provide clinically valid estimates of their effects. Apart from this network meta-analysis, no review has been published to investigate the efficacies of multiple treatments in inducing ovulation in CCR-PCOS patients using a predefined treatment duration of between 3 and 6 cycles. Here, we did not perform a cost-effectiveness analysis because most of the drugs were off patent and produced by different companies in generic form, whose costs varied largely. Furthermore, the costs of drugs were minimal compared to that of LOD surgery, whose long-term effects need further exploring.

In conclusion, this review and network meta-analysis demonstrated that there was still insufficient high-quality evidence to detect the most effective therapy for ovulation-induction in CCR-PCOS patients. But the results of the ranking probabilities showed that, apart from hMG and FSH, metformin+letrozole was potentially more effective than other treatments. But this tentative finding should be substantiated and strengthened by high quality evidence. What was more, therapies should be individualized based on local facilities, physical signs as well as patient preference^49–51^.

## Methods

### Literature Search

The study selection process involved the performance of both electronic and manual searching. We searched the Cochrane Library, PubMed, and EMBASE on September 1, 2016 to identify randomized clinical studies investigating any of the 9 clinically widely used therapies (CC, metformin, letrozole, FSH, hMG, metformin+CC, metformin+letrozole, ULOD and BLOD) for the treatment of CCR PCOS. We only included studies published in English, and there was no limitation on publication year. The latest search was updated on January 20, 2017. After the primary electronic search, we scrutinized the reference lists of relevant systematic reviews and meta-analyses to identify potentially missed studies.

### Study Selection

Two authors (Yu and Fang) independently conducted the study selection process. In general, we specifically targeted randomized controlled trials comparing at least 2 of the aforementioned 9 mono-ovulation therapies as a mono-therapy over the course of a period ranging between 3 and 6 months. Discrepancies were resolved via discussion. First, we excluded the following types of studies by reviewing titles and abstracts after removing duplicates: conference abstracts, reviews and meta-analyses, non-randomized trials, trials with non-CCR PCOS participants, trials comparing different dosages, and trials comparing different treatment durations. Second, we reviewed full texts to exclude trials conducted using crossover therapies from which data for mono-therapies could not be extracted; we also excluded trials comparing 1 of the aforementioned 9 therapies with placebo therapy. Third, included trials had to report at least 1 of the following 5 reproductive outcomes: ovulation rate, pregnancy rate, live birth rate, abortion rate and multiple pregnancy rate. Finally, trials with a follow-up period of less than 3 and more than 6 cycles were excluded.

### Data Extraction

Two authors extracted information from the included trials for their locations; treatment durations; criteria for CCR; interventions; dosages; assisted reproductive therapies; number of participants/cycles; participant ages, body mass indices, and reproductive outcomes (number of ovulation cycles and number of patients who became pregnant, aborted, gave birth and conceived multiples); and the occurrence of OHSS and side effects. Discrepancies were solved via discussion or referred to the corresponding author.

### Quality Assessment

The biases of included trials were assessed according to the Cochrane Handbook for Systematic Reviews of Interventions Version 5.3 with respect to the following aspects: sequence generation, allocation concealment, blinding, incomplete outcome data, selective outcome reporting and other bias^52^. Funnel plots were created to make visual assessments of publication bias. The four-step approach newly developed by the Grading of Recommendations Assessment, Development and Evaluation framework (GRADE) working group was used to rate the quality of evidence in each of the direct, indirect and network estimates^53, 54^. The inconsistencies between direct and indirect comparisons were assessed using loop-specific heterogeneity tests in Stata 14.0. The quality rating started with high because all included trials were randomized trials. The quality rating would be rated down by -1 (serious concern) or -2 (very serious concern) for the following reasons; limitations in study design (failure to conceal random allocation or lack of blinding), inconsistency (significant heterogeneities across trials in comparisons), indirectness (differences in patient characteristics, co-interventions and measurements of outcomes), imprecision (number of events, -1 if less than 300, -2 if less than 50) and publication bias. Based on the above principles, the direct estimates were provided by head-to-head comparisons and indirect estimates were based on two direct estimates (A *v* C and B *v* C for A *v* B). The lower rating of two direct estimates constitutes the rating of the indirect comparison. Finally, the higher rating of the direct and indirect evidence was regarded as the quality rating for the network meta-analysis^53, 54^.

### Data Synthesis and Statistical Analysis

In this network meta-analysis, the endpoints we focused on included reproductive outcomes (pregnancy rates per ITT, live birth rates per ITT, abortion rates per pregnancy, ovulation rates per cycle, and multiple pregnancy rates per pregnancy) and adverse events (side effects and the occurrence of OHSS). All outcomes were assessed as dichotomous variables. Pregnancy and live birth rates were evaluated based on their count per ITT regardless of how the original trials were analysed. When dealing with the missing participants, we assumed that they did not respond to the treatment. We defined the treatment duration as ranging between 3 and 6 cycles, extracted data only from that period, and then entered these data into a spreadsheet.

First, we performed traditional pairwise meta-analyses using Stata 14.0 software. Heterogeneity across trials was measured using the I^2^ statistic^52^. An I^2^ > 50% indicated significant heterogeneity and a random effects model was used; otherwise, a fixed effects model was used. The results are presented as ORs with 95% confidence intervals (CI), and *p* < 0.05 indicated statistical significance.

Second, we performed the network meta-analysis using the Markov Chains Monte Carlo (MCMC) method based on the Bayesian framework and using the non-programming software of the Aggregate Data Drug Information System (ADDIS) version 1.16.7. In the Bayesian inference, the posterior distribution was calculated by combining information about prior distributions and observed data. The model implemented in ADDIS used MCMC methods to generate samples from the posterior distribution of the model. Before conducting the network meta-analysis, consistency and convergence were assessed. We used the node splitting method to verify inconsistencies, which separated evidence for a particular comparison into direct and indirect evidence, and *p* < 0.05 indicated a significant inconsistency. If no significant inconsistency was identified, the relative effects of the interventions were analysed using a consistency model; otherwise, an inconsistency model was used^55^. Convergence was assessed using the Brooks-Gelman-Rulin method^53^. If the potential scale reduction factors (PSRF) were close to 1 for all of the chains, the results were considered to be well-converged. The ranking of all evaluated treatments was simultaneously performed using the Bayesian approach. The calculation of ranking probabilities allowed us to estimate the potential efficacy of one treatment relative to that of another, within both rank-over-treatments and treatment-over-ranks analyses.

## Electronic supplementary material

Supplementary file
