# Supplementary material for: Comparative effectiveness of 9 ovulation-induction therapies in patients with clomiphene citrate-resistant polycystic ovary syndrome: a network meta-analysis
Source: Sci Rep. 2017 Jun 19;7:3812. doi: 10.1038/s41598-017-03803-9 (PMC5476620; doi:10.1038/s41598-017-03803-9)
Supplement: Supplementary file 1 — Supplementary file [file 41598_2017_3803_MOESM1_ESM.pdf]

Comparative effectiveness of 9 ovulation-induction therapies in patients with clomiphene citrate-resistant polycystic ovary syndrome: a network meta- analysis

Yiping Yu [a†], Lanlan Fang [a†], Ruizhe Zhang[a], Jingyan He[a], Yujing Xiong[a], Xiaoyi Guo[a], Qingyun Du[a], Yan Huang[a] and Yingpu Sun[a]*

[a] *Department of Reproductive Medical Center, First Affiliated Hospital of Zhengzhou University, China*

** Corresponding author: Ying-pu Sun*

Full postal address: Jianshe Dong Road, Erqi District, Zhengzhou City, Henan Province, P.R.China

Tel.: +86 0371 66913635

Fax: +86 371 67966161

E-mail address: syp2008@vip.sina.com

Supplementary Table S1. Characteristics of included trials and patients.

	Study (year)	Country	Duration (months)	Intervention	Dosages	Assisted reproductive therapies	Num. of Participants (patients/cycles)	Age (years old)	BMI （kg/m2）	Num. of ovulation cycles	Num. of pregnancies	Num. of live births	Num. of abortions	Num. of multiple pregnancies	Num. of OHSS	Num. of adverse effects
1	Abd Elgafor 2013	Egypt	6	MET+LE	MET (850→1700mg)+ LE (5mg,d3-7)	timed intercourse	73/-	24.7±1.8	31.5±3.3	35^	26	-	4	-	-	-
				BLOD			73/-	25.1±2.1	32.4±4.4	34^	23	-	3	-	-	-
2	Abdellah 2011	Egypt	6	LE	5mg, d3-5	timed intercourse	74/346	23.9±3.2	27.3±2.6	204	25	23	2	0	0	-
				BLOD			73/373	23.6±3.2	27.1±2.6	177	20	16	4	0	0	-
3	Abu Hashim 2010a	Egypt	3	MET+CC	MET (1500mg, 6-8 weeks ahead)+ CC (100mg→150mg,d3-7)	timed intercourse	75/205	27.5±2.4	26.4±3.3	127	18	14	4	2	-	6
				FSH	from 75IU increased by 37.5IU		78/186	26.8±2.2	26.3±3.6	156	32	27	5	6	-	0
4	Abu Hashim 2010b	Egypt	6	LE	2.5mg,d3-7	timed intercourse	128/512	27.3±2.6	26.4±3.3	335	36	32	4	0	0	-
				BLOD			132/525	26.4±2.4	26.6±3.6	364	37	33	4	0	0	-
5	Abu Hashim 2010c	Egypt	3	LE	2.5mg,d3-7	timed intercourse	123/285	28.3±2.7	29.1±3.2	185	39	35	4	0	0	0
				MET+CC	MET (1500mg, 6-8 weeks ahead)+ CC (100mg→150mg,d3-7)		127/297	26.2±2.2	30.1±2.3	207	42	38	4	3	0	10

6	Abu Hashim 2011a	Egypt	6	MET+CC	MET (1500mg, 6-8 weeks ahead)+CC (100mg→150mg,d3-7)	timed intercourse	138/576	27.2±2.5	26.2±3.4	386	40	-	8	4	0	13
				BLOD			144/558	26.5±2.3	26.1±3.5	381	43	-	9	0	0	0
7	Abu Hashim 2011b	Egypt	6	BLOD		timed intercourse	87/-	26.3±2.6	24.7±3.3	-	34	28	6	0	0	-
				CC	50-150mg,d3-7		89/-	25.2±2.4	25.4±3.6	-	30	25	5	4	0	-
8	Balen 1994	United Kingdom	3	ULOD		timed intercourse	4	-	-	3^	-	-	-	-	-	-
				BLOD			6	-	-	2^	-	-	-	-	-	-
9	Begum# 2013	Bangladesh	6	MET+CC	MET(1500mg, 4weeks ahead)+CC(150mg,d3-7)	timed intercourse	55/-	27.0±4.1	27.71±3.61	15^	7	5	1	0	0	0
				FSH	75IU		55/-	27.2±4.2	28.36±4.54	41^	16	12	3	0	0	0
10	Davar 2011	Iran	3	MET+CC	MET(1500mg,6-8 weeks ahead)+CC(150mg,d3-7)	IUI	48/78	29.5±3.5	29.21±2.92	-	1	-	1	-	-	0
				MET+LE	MET(1500mg,6-8 weeks ahead)+ LE(5.0mg,d3-7)		50/70	28.5±3.1	28.98±3.83	-	4	-	0	-	-	2
11	Farquhar 2002	New Zealand	6	BLOD		timed intercourse	29/-	26.3±4.3	27.4±4.2	-	8	4	4	-	0	0
			3	FSH	from 75IU on		21/-	25.6±4.1	26.3±3.2	-	7	4	3	-	0	1
12	George 2003	India	3	MET+CC	MET(1500mg,6 months ahead)+CC(150mg,d3-7)	timed intercourse	30/-	25.1±3.0	25.5±3.7	14^	5	-	-	-	-	3
				hMG	75IU increased by 75IU		30/50	26±2.9	24.6±2.6	-	7	-	-	-	-	0
13	Hamed 2010	Egypt	6	diagnostic LOD+MET	1700mg	timed intercourse	55/281	23.6±2.6	35.6±4.4	94	11	9	2	-	-	-
				BLOD			55/258	24.3±4.5	36.1±3.6	131	21	17	4	-	-	-

14	Kaya 2005	Turkey	6	BLOD		timed intercourse	17/-	29.6±4.7	28.3±3.9	-	6	-	-	0	0	0
				FSH	Step-up protocol		18/-	29.6±4.2	27.8±4.8	-	6	-	-	2	4	0
15	Liu 2015	China	6	LE	2.5mg,d5-9	timed intercourse	71/382	29.50±3.26	22.50±1.46	305	29	27	2	1	0	0
				BLOD			70/358	28.08±3.61	22.41±2.07	237	19	16	3	0	0	0
16	Malkawi 2002	Jordan	6	MET+CC	MET (1700mg)+CC (50→200mg, d5-9)	timed intercourse	16/63	29±3.1	27.5±4.1	43	9	-	-	-	0	-
				CC	CC (50→200mg, d2-6)		12/66	29±7.3	27.8±3.3	16	2	-	-	-	2	-
17	McFaul 1990	Northern Ireland	6	FSH	from 150IU increased by 150IU	timed intercourse	34/68	-	29.3	-	6	6	1	0	-	-
				hMG	75IU		15/41	-	28.4	-	5	3	0	1	-	-
18	Mehrabian* 2012	Iran	3	BLOD		timed intercourse	52/-	29.17±5.47	27.73±6.16	-	18	-	5	1	0	-
				hMG	-		52/-	28.51±5.51	27.55±6.07	-	37	-	6	5	2	-
19	Palomba 2004	Italy	6	MET	1700mg	timed intercourse	60/210	26.8±2.2	28.1±1.7	115	39	32	6	0	-	12
				BLOD			60/231	27.5±2.4	27.6±1.6	123	31	30	9	0	-	3
20	Palomba 2010	Italy	6	BLOD		timed intercourse	25/92	28.2±4.3	29.8±3.2	52	14	13	2	0	0	0
				MET+CC	MET(850mg-1700mg for 6-8weeks)+ CC (150mg,d3-7)		25/107	27.5±4.8	30.2±3.4	77	12	12	2	0	0	4

21	Rezk 2016	Egypt	6	ULOD		timed intercourse	54/-	29.7±1.5	23.9±2.1	17^	6	-	-	-	-	-
				BLOD			54/-	29.8±1.4	24.4±1.8	31^	26	-	-	-	-	-
22	Roy* 2009	India	3	ULOD		timed intercourse	22/-	28.2±1.7	-	8^	7	8	2	0	-	-
				BLOD			22/-	28.8±2.9	-	10^	4	8	2	0	-	-
23	Sharma* 2006	India	3	ULOD		timed intercourse	10/-	27.3 (21–32)	-	6^	5	4	1	-	-	-
				BLOD			10/-	25.5 (23–30)	-	8^	6	6	0	-	-	-
24	Sohrabvand 2006	Iran	3	MET+LE	MET(1500mg, 6-8 weeks ahead)+ LE (2.5mg,d3-7)	timed intercourse	29/53	28.24±3.11	29.98±4.83	48	10	10	0	-	-	-
				MET+CC	MET(1500mg,6-8weeks ahead)+ CC (100mg,d3-7)		30/67	29.55±3.47	30.21±3.92	22	5	3	2	-	-	-
25	Vandermolen 2001	America	6	MET+CC	MET(1500mg,7weeks ahead)+CC (50→100mg, for 5 days)	timed intercourse	12/-	29±1.2	37.6±4.3	9^	6	4	2	0	0	0
				CC	50mg→150mg for 5 days		15/-	30±1.0	38.4±2.2	4^	1	1	0	0	0	0
26	Youssef 2007	Egypt	6	ULOD		timed intercourse	43/-	18.3±3.5	26.1±1.9	34^	18	-	4	-	-	-
				BLOD			44/-	23.5±2.5	23.5±2.5	34^	17	-	4	-	-	-

CC: clomiphene citrate, MET: metformin, LE: letrozole, FSH: follicle-stimulating hormone, hMG: human menopausal gonadotropin, MET+CC: metformin combined with clomiphene citrate, MET+LE: metformin combined with letrozole, ULOD: unilateral laparoscopic ovarian drilling and BLOD: bilateral laparoscopic ovarian drilling. #: data was extracted from two of the original three arms. * if anovulation persisted 3 cycles after ULOD/BLOD, CC or gonadotropins would applied and the data used was extracted from the first 3 cycles without administration of ovulation-induction drugs. ^: the number of ovulation cycles was unavailable; data shown was the number of people who ovulated; IUI: intrauterine insemination; BMI: body mass index; d3-7: from day 3 to day 7 on menstrual cycles and by analogy.

	Random sequence generation (selection bias)	Allocation concealment (selection bias)	Blinding of participants and personnel (performance bias)	Blinding of outcome assessment (detection bias)	Incomplete outcome data (attrition bias)	Selective reporting (reporting bias)	Other bias
Abd Elgafor,2013	+	+	−	?	?	?	?
Abdellah, 2011	+	+	−	−	+	?	?
Abu Hashim,2010a	+	+	−	+	+	?	?
Abu Hashim,2010b	+	+	+	+	+	?	?
Abu Hashim,2010c	+	+	−	+	+	?	?
Abu Hashim,2011a	+	+	−	−	+	?	?
Abu Hashim,2011b	+	+	−	+	+	?	?
Balen,1994	?	?	−	+	−	?	?
Begum,2013	+	?	?	?	−	?	?
Davar,2011	+	?	−	−	+	?	?
Farquhar,2002	+	+	−	+	+	?	?
George,2003	+	+	?	?	−	?	?
Hamed,2010	+	+	?	+	+	?	?
Kaya,2005	+	+	−	−	+	?	?
Liu,2015	+	−	−	+	+	?	?
Malkawi,2002	+	?	?	?	+	?	?
McFaul,1990	?	?	−	−	+	?	?
Mehrabian,2012	+	−	−	+	+	?	?
Palomba,2004	+	?	+	+	+	?	?
Palomba,2010	+	+	−	+	+	?	?
Rezk,2016	+	?	?	?	+	?	?
Roy,2009	?	?	−	−	+	?	?
Sharma,2009	+	?	−	−	+	?	?
Sohrabvand,2006	+	?	−	+	+	?	?
Vandermolen,2001	+	+	+	+	+	?	?
Youssef,2007	?	+	+	+	+	?	?

Supplementary Figure S1. Summary of risk of bias of included trials.

+ : low risk; ? : unclear risk; − : high risk.

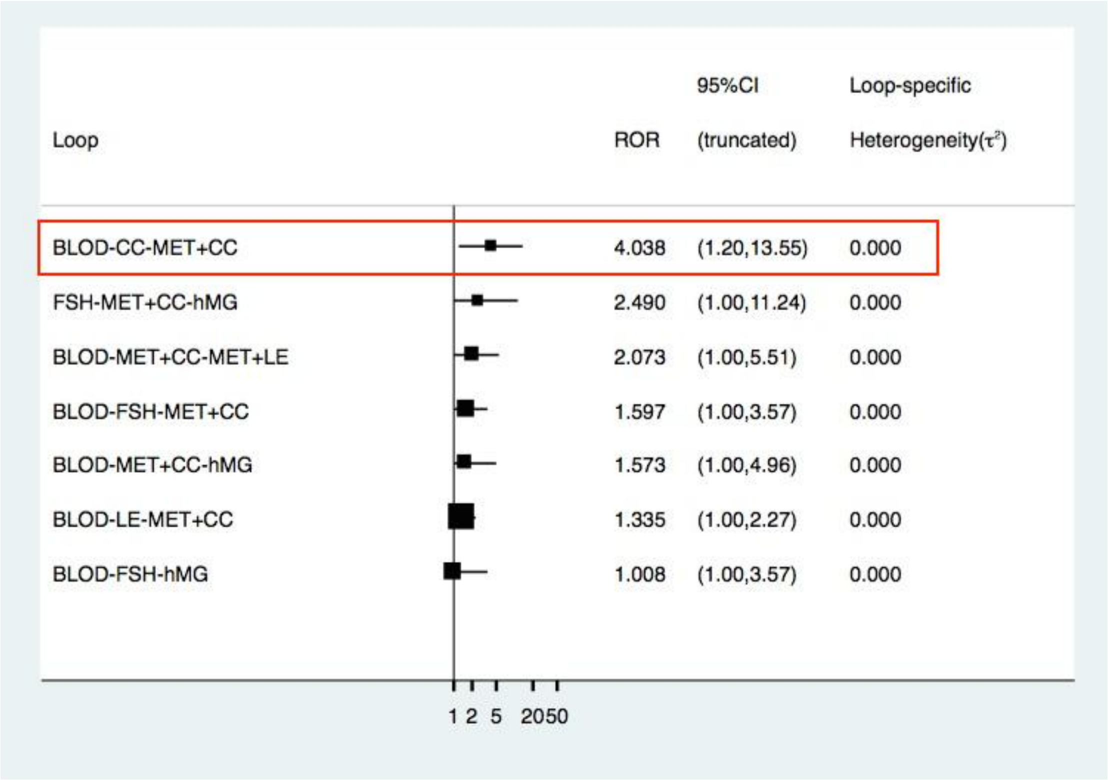

Supplementary Figure S2. Loop-specific heterogeneity test of pregnancy rate. ROR higher than 1 indicates significant heterogeneity in closed loop. CC: clomiphene citrate, MET: metformin, LE: letrozole, FSH: follicle-stimulating hormone, hMG: human menopausal gonadotropin, MET+CC: metformin combined with clomiphene citrate, MET+LE: metformin combined with letrozole, ULOD: unilateral laparoscopic ovarian drilling, BLOD: bilateral laparoscopic ovarian drilling. Significant heterogeneity might exist in BLOD-CC-MET+CC loop.

Supplementary Table S2. Quality ratings for comparisons of therapies to induce ovulation in CCR-PCOS patients.

Direct Comparisons	Direct Comparisons	Trials included	Decrease quality of evidence							Quality
			Limitations in design			inconsistency	indirectiness	imprecision	publication bias	
			allocation concealment	random sequence generation	blinding					
BLOD vs. CC	1	Abu Hashim,2011b	0	0	-1	0	0	-1	0	low
BLOD vs. FSH	2	Farquhar,2002<br>Kaya,2005	0	0	-1	0	0	-2	0	very low
BLOD vs. LE	3	Abdellah,2011<br>Abu Hashim,2010b<br>Liu,2015	-1	0	-1	0	0	-1	0	low
BLOD vs. MET	2	Palomba, 2004<br>Hamed,2010	0	0	-1	-1	0	-1	0	very low
BLOD vs. MET+CC	2	Abu Hashim,2011a<br>Palomba, 2010	0	0	-1	0	0	-1	0	low
BLOD vs. ULOD	5	Balen,1994<br>Rezk,2016<br>Roy,2009<br>Sharma,2006<br>Youssef,2007	0	0	-1	-1	0	-1	-1	very low
BLOD vs.	1	Mehrabian,2012	-1	0	-1	0	0	-1	0	low

hMG										
BLOD vs. MET+LE	1	Abd Elgafor,2013	0	0	-1	0	0	-1	0	low
CC vs. MET+CC	2	Malkawi,2002<br>Vandermolen,2001	0	0	0	0	0	-2	0	low
FSH vs. MET+CC	2	Abu Hashim,2010a<br>Begum,2013	0	0	-1	0	0	-1	0	low
FSH vs. hMG	1	McFaul,1990	0	0	-1	0	0	-2	0	very low
LE vs. MET+CC	1	Abu Hashim,2010c	0	0	-1	0	0	-1	0	low
MET+CC vs. hMG	1	George,2003	0	0	0	0	0	-2	-1	very low
MET+CC vs. MET+LE	2	Davar,2011<br>Sohrabvand，2006	0	0	-1	0	0	-2	0	very low
Indirect Comparisons	Loops for indirect estimate	Trials included								
BLOD vs. CC	BLOD-MET+CC-CC	Abu Hashim,2011a<br>Palomba, 2010<br>Malkawi,2002<br>Vandermolen,2001	0	0	-1	-1	0	-1	0	very low
BLOD vs. FSH	BLOD-MET+CC-FSH	Abu Hashim,2011a<br>Palomba, 2010<br>Begum,2013	0	0	-1	0	0	-1	0	low

		Abu Hashim,2010a								
BLOD vs. LE	BLOD-MET+CC-LE	Abu Hashim,2011a<br>Palomba, 2010<br>Abu Hashim,2010c	0	0	-1	0	0	-1	0	low
BLOD vs. MET	/	/	/	/	/	/	/	/	/	/
BLOD vs. MET+CC	BLOD-LE-MET+CC	Abdellah,2011<br>Abu Hashim,2010b<br>Liu,2015<br>Abu Hashim,2010a<br>Begum,2013	-1	0	-1	0	0	-1	0	low
BLOD vs. ULOD	/	/	/	/	/	/	/	/	/	/
BLOD vs. hMG	BLOD-MET+CC-hMG	George,2003<br>Abu Hashim,2011a<br>Palomba, 2010	0	0	-1	0	0	-1	0	low
BLOD vs. MET+LE	BLOD-MET+CC-MET+LE	Abu Hashim,2011a<br>Palomba, 2010<br>Davar,2011<br>Sohrabvand，2006	0	0	-1	0	0	-1	0	low
CC vs. MET+CC	CC-BLOD-MET+CC	Abu Hashim,2011b<br>Abu Hashim,2011a<br>Palomba, 2010	0	0	-1	-1	0	-1	0	very low
FSH vs. MET+CC	FSH-BLOD-MET+CC	Farquhar,2002<br>Kaya,2005	0	0	-1	0	0	-1	0	low

		Abu Hashim,2011a<br>Palomba, 2010								
FSH vs. hMG	FSH-BLOD-hMG	Farquhar,2002<br>Kaya,2005<br>Mehrabian，2012	-1	0	-1	0	0	-1	0	low
LE vs. MET+CC	LE-BLOD-MET+CC	Abdellah,2011<br>Abu Hashim,2010b<br>Liu,2015<br>Abu Hashim,2011a<br>Palomba, 2010	-1	0	-1	0	0	-1	0	low
MET+CC vs. hMG	MET+CC-BLOD-hMG	Abu Hashim,2011a<br>Palomba, 2010<br>Mehrabian，2012	-1	0	-1	0	0	-1	0	low
MET+CC vs. MET+LE	MET+CC-BLOD-MET+LE	Abu Hashim,2011a<br>Palomba, 2010<br>Abd Elgafor,2013	0	0	-1	0	0	-1	0	low
CC vs. FSH	CC-MET+CC-FSH	Abu Hashim,2010a<br>Begum,2013<br>Malkawi,2002<br>Vandermolen,2001	0	0	-1	0	0	-1	0	low
CC vs. LE	CC-BLOD-LE	Abdellah,2011<br>Abu Hashim,2010b<br>Abu Hashim,2011b	0	0	-1	0	0	-1	0	low
CC vs. MET	CC-BLOD-MET	Abu Hashim,2011b<br>Palomba, 2004	0	0	-1	0	0	-1	0	low

		Hamed,2010								
CC vs. ULOD	CC-BLOD-ULOD	Abu Hashim,2011b<br>Rezk,2016<br>Roy,2009<br>Sharma,2006<br>Youssef,2007<br>Balen,1994	0	0	-1	-1	0	-1	-1	very low
CC vs. hMG	CC-MET+CC-hMG	Malkawi,2002<br>Vandermolen,2001<br>George,2003	0	0	0	0	0	-2	-1	very low
CC vs. MET+LE	CC-MET+CC-MET+LE	Malkawi,2002<br>Vandermolen,2001<br>Davar,2011<br>Sohrabvand，2006	0	0	-1	0	0	-2	0	very low
FSH vs. LE	FSH-BLOD-LE	Farquhar,2002<br>Kaya,2005<br>Abdellah,2011<br>Abu Hashim,2010b Liu,2015	-1	0	-1	0	0	-1	0	low
FSH vs. MET	FSH-BLOD-MET	Farquhar,2002<br>Kaya,2005<br>Palomba, 2004<br>Hamed,2010	0	0	-1	0	0	-1	0	low
FSH vs. ULOD	FSH-BLOD-ULOD	Farquhar,2002<br>Kaya,2005<br>Rezk,2016	0	0	-1	-1	0	-1	-1	very low

		Roy,2009<br>Sharma,2006<br>Youssef,2007<br>Balen,1994								
FSH vs. MET+LE	FSH-MET+CC-MET+LE	Abu Hashim,2010a<br>Begum,2013<br>Davar,2011<br>Sohrabvand，2006	0	0	-1	0	0	-1	0	low
LE vs. MET	LE-BLOD-MET	Abdellah,2011<br>Abu Hashim,2010b<br>Liu,2015<br>Palomba, 2004<br>Hamed,2010	-1	0	-1	-1	0	-1	0	low
LE vs. ULOD	LE-BLOD-ULOD	Abdellah,2011<br>Abu Hashim,2010b<br>Liu,2015<br>Rezk,2016<br>Roy,2009<br>Sharma,2006<br>Youssef,2007<br>Balen,1994	-1	0	-1	-1	0	-1	-1	very low
LE vs. hMG	LE-BLOD-hMG	Abdellah,2011<br>Abu Hashim,2010b<br>Liu,2015<br>Mehrabian，2012	-1	0	-1	0	0	-1	0	low

LE vs. MET+LE	LE-BLOD-MET+LE	Abdellah,2011<br>Abu Hashim,2010b<br>Liu,2015<br>Abd Elgafor,2013	-1	0	0	0	0	-1	0	low
MET vs. MET+CC	MET-BLOD-MET+CC	Palomba, 2004<br>Hamed,2010<br>Abu Hashim,2011a<br>Palomba, 2010	0	0	-1	-1	0	-1	0	very low
MET vs. ULOD	MET-BLOD-ULOD	Palomba, 2004<br>Hamed,2010<br>Rezk,2016<br>Roy,2009<br>Sharma,2006<br>Youssef,2007<br>Balen,1994	0	0	-1	-1	0	-1	-1	very low
MET vs. hMG	MET-BLOD-hMG	Palomba, 2004<br>Hamed,2010<br>Mehrabian, 2012	-1	0	-1	0	0	-1	0	low
MET vs. MET+LE	MET-BLOD-MET+LE	Palomba, 2004<br>Hamed,2010<br>Abd Elgafor,2013	0	0	-1	-1	0	-1	0	very low
MET+CC vs. ULOD	MET+CC-BLOD-ULOD	Abu Hashim,2011a<br>Palomba, 2010<br>Rezk,2016<br>Roy,2009	0	0	-1	-1	0	-1	-1	very low

		Sharma,2006<br>Youssef,2007<br>Balen,1994								
ULOD vs. hMG	ULOD-BLOD-hMG	Rezk,2016<br>Roy,2009<br>Sharma,2006<br>Youssef,2007<br>Balen,1994<br>Mehrabian，2012	-1	0	-1	-1	0	-1	-1	very low
ULOD vs. MET+LE	ULOD-BLOD-MET+LE	Rezk,2016<br>Roy,2009<br>Sharma,2006<br>Youssef,2007<br>Balen,1994<br>Abd Elgafor,2013	0	0	-1	-1	0	-1	-1	very low
hMG vs. MET+LE	hMG-BLOD-MET+LE	Mehrabian，2012<br>Abd Elgafor,2013	-1	0	-1	0	0	-1	0	low

CC: clomiphene citrate, MET: metformin, LE: letrozole, FSH: follicle-stimulating hormone, hMG: human menopausal gonadotropin, MET+CC: metformin combined with clomiphene citrate, MET+LE: metformin combined with letrozole, ULOD: unilateral laparoscopic ovarian drilling, BLOD: bilateral laparoscopic ovarian drilling. If num. of events was less than 50, there was very serious imprecision (-2); if num. of events was between 50 and 300, there was serious imprecision (-1); if num. of events was more than 300, there was no imprecision (0). “/”: could not be estimated.

Supplementary Table S3. Results for sensitivity analyses by excluding each of the trials.

Table S3 a: Sensitivity analyses for pregnancy rate

Trials excluded \ Ranking	1	2	3	4	5	6	7	8	9
Abd Elgafor,2013	MET+LE	hMG	FSH	MET+CC	LE	BLOD	MET	ULOD	CC
Abdellah,2011	hMG	MET+LE	FSH	LE	MET+CC	BLOD	MET	ULOD	CC
Abu Hashim,2010a	hMG	MET+LE	FSH	LE	MET+CC	BLOD	MET	ULOD	CC
Abu Hashim,2010b	hMG	MET+LE	FSH	LE	MET+CC	BLOD	MET	ULOD	CC
Abu Hashim,2010c	hMG	MET+LE	FSH	LE	MET+CC	BLOD	MET	ULOD	CC
Abu Hashim,2011a	hMG	MET+LE	FSH	LE	MET+CC	BLOD	MET	ULOD	CC
Abu Hashim,2011b	hMG	MET+LE	FSH	MET+CC	LE	BLOD	MET	ULOD	CC
Balen,1994	hMG	MET+LE	FSH	LE	MET+CC	BLOD	MET	ULOD	CC
Begum,2013	hMG	MET+LE	FSH	LE	MET+CC	BLOD	MET	ULOD	CC
Davar,2011	hMG	MET+LE	FSH	LE	MET+CC	BLOD	MET	ULOD	CC
Farquhar,2002	hMG	MET+LE	FSH	LE	MET+CC	BLOD	MET	ULOD	CC
George,2003	hMG	MET+LE	FSH	LE	MET+CC	BLOD	MET	ULOD	CC
Hamed,2010	hMG	FSH	MET+LE	LE	MET	BLOD	MET+ CC	ULOD	CC
Kaya,2005	hMG	FSH	MET+LE	LE	MET+CC	BLOD	MET	ULOD	CC
Liu,2015	hMG	MET+LE	FSH	MET+CC	LE	BLOD	MET	ULOD	CC
Malkawi,2002	hMG	MET+LE	FSH	LE	BLOD	MET+CC	MET	ULOD	CC
McFaul,1990	hMG	FSH	MET+LE	LE	MET+CC	BLOD	MET	ULOD	CC
Mehrabian，2012	hMG	MET+LE	FSH	LE	BLOD	MET+CC	MET	ULOD	CC
Palomba, 2004	hMG	MET+LE	FSH	LE	MET+CC	BLOD	MET	ULOD	CC
Palomba, 2010	hMG	MET+LE	FSH	MET+CC	LE	BLOD	MET	ULOD	CC
Rezk,2016	hMG	FSH	MET+LE	LE	MET+CC	BLOD	MET	ULOD	CC
Roy,2009	hMG	MET+LE	FSH	LE	MET+CC	BLOD	MET	ULOD	CC
Sharma,2006	hMG	MET+LE	FSH	LE	MET+CC	BLOD	MET	ULOD	CC
Sohrabvand，2006	hMG	MET+LE	FSH	MET+CC	LE	BLOD	MET	ULOD	CC
Vandermolen,2001	hMG	MET+LE	FSH	LE	BLOD	MET+CC	MET	ULOD	CC
Youssef,2007	hMG	MET+LE	FSH	LE	MET+CC	BLOD	MET	ULOD	CC

Table S3 b: Sensitivity analyses for live birth rate

Trials excluded \ Ranking	1	2	3	4	5	6	7	8	9
Abdellah, 2011	MET+LE	hMG	FSH	MET+CC	LE	BLOD	MET	ULOD	CC
Abu Hashim,2010a	MET+LE	hMG	FSH	LE	MET+CC	BLOD	MET	ULOD	CC
Abu Hashim,2010b	MET+LE	hMG	FSH	LE	MET+CC	BLOD	MET	ULOD	CC
Abu Hashim,2010c	MET+LE	hMG	FSH	LE	MET+CC	BLOD	MET	ULOD	CC

Abu Hashim,2011b	MET+LE	hMG	FSH	LE	MET+CC	BLOD	MET	ULOD	CC
Begum,2013	MET+LE	hMG	FSH	MET+CC	LE	BLOD	MET	ULOD	CC
Farquhar,2002	MET+LE	hMG	FSH	MET+CC	LE	BLOD	MET	ULOD	CC
Hamed,2010	MET+LE	hMG	FSH	MET	LE	MET+CC	BLOD	ULOD	CC
Liu,2015	MET+LE	hMG	FSH	MET+CC	LE	BLOD	MET	ULOD	CC
McFaul,1990	MET+LE	FSH	MET+CC	LE	BLOD	MET	ULOD	CC	/
Palomba, 2004	MET+LE	hMG	FSH	MET+CC	LE	BLOD	ULOD	CC	MET
Palomba, 2010a	MET+LE	hMG	FSH	MET+CC	LE	BLOD	MET	ULOD	CC
Roy,2009	MET+LE	hMG	FSH	MET+CC	LE	BLOD	MET	CC	ULOD
Sharma,2006	MET+LE	hMG	FSH	MET+CC	LE	BLOD	MET	ULOD	CC
Sohrabvand，2006	hMG	FSH	MET+CC	LE	BLOD	MET	ULOD	CC	/
Vandermolen,2001	MET+LE	hMG	FSH	LE	MET+CC	BLOD	MET	CC	ULOD

Table S3 c: Sensitivity analyses for ovulation rate.

Ranking<br>Trials excluded	1	2	3	4	5	6	7
Abdellah,2011	MET+LE	FSH	MET+CC	LE	BLOD	MET	CC
Abu Hashim,2010a	MET+LE	MET+CC	LE	BLOD	MET	CC	/
Abu Hashim,2010b	MET+LE	FSH	LE	MET+CC	BLOD	MET	CC
Abu Hashim,2010C	MET+LE	FSH	MET+CC	LE	BLOD	MET	CC
Abu Hashim,2011a	MET+LE	FSH	MET+CC	LE	BLOD	MET	CC
Hamed,2010	MET+LE	FSH	MET+CC	LE	BLOD	MET	CC
Liu,2015	MET+LE	FSH	MET+CC	LE	BLOD	MET	CC
Malkawi,2002	MET+LE	FSH	MET+CC	LE	BLOD	MET	/
Palomba, 2004	MET+LE	FSH	MET+CC	LE	BLOD	MET	CC
Palomba, 2010	MET+LE	FSH	MET+CC	LE	BLOD	MET	CC
Sohrabvand，2006	FSH	MET+CC	LE	BLOD	MET	CC	/

Table S3 d: Sensitivity analyses for abortion rate.

Ranking<br>Trials excluded	1	2	3	4	5	6	7	8	9
Abd Elgafor,2013	ULOD	CC	BLOD	MET+CC	LE	MET	FSH	hMG	MET+LE
Abdellah, 2011	MET+CC	FSH	ULOD	BLOD	LE	CC	MET	hMG	MET+LE
Abu Hashim,2010a	FSH	ULOD	MET+CC	BLOD	CC	LE	MET	hMG	MET+LE
Abu Hashim,2010b	ULOD	MET+CC	BLOD	CC	MET	FSH	hMG	LE	MET+LE
Abu Hashim,2010c	MET+CC	FSH	BLOD	ULOD	CC	LE	hMG	MET	MET+LE
Abu Hashim,2011a	MET+CC	FSH	ULOD	BLOD	LE	CC	MET	hMG	MET+LE
Abu Hashim,2011b	ULOD	MET+CC	BLOD	FSH	LE	MET	hMG	MET+LE	CC

Begum,2013	MET+CC	ULOD	FSH	BLOD	LE	MET	CC	hMG	MET+LE
Davar,2011	ULOD	MET+CC	BLOD	LE	FSH	CC	MET	MET+LE	hMG
Farquhar,2002	FSH	MET+CC	BLOD	ULOD	LE	MET	CC	hMG	MET+LE
Hamed,2010	MET+CC	FSH	ULOD	BLOD	LE	CC	MET	hMG	MET+LE
Liu,2015	MET+CC	FSH	ULOD	BLOD	CC	LE	MET	hMG	MET+LE
McFaul,1990	MET+CC	ULOD	FSH	BLOD	LE	CC	MET	hMG	MET+LE
Mehrabian，2012	ULOD	MET+CC	FSH	BLOD	LE	CC	MET	MET+LE	hMG
Palomba, 2004	MET	MET+CC	ULOD	FSH	BLOD	LE	CC	hMG	MET+LE
Palomba, 2010a	ULOD	MET+CC	BLOD	CC	LE	MET	FSH	MET+LE	hMG
Roy,2009	ULOD	MET+CC	BLOD	FSH	LE	MET	CC	hMG	MET+LE
Sharma,2006	MET+CC	FSH	CC	BLOD	LE	CC	MET	hMG	MET+LE
Sohrabvand, 2006	ULOD	MET+CC	CC	BLOD	FSH	LE	MET	MET+LE	hMG
Vandermolen,2001	MET+CC	FSH	BLOD	CC	ULOD	LE	MET	hMG	MET+LE
Youssef,2007	ULOD	MET+CC	BLOD	FSH	CC	LE	MET	hMG	MET+LE

Table S3 e: Sensitivity analyses for multiple pregnancies.

Ranking / Trials excluded	1	2	3	4	5	6
Abu Hashim,2010a	MET+CC	LE	CC	hMG	FSH	BLOD
Abu Hashim,2010c	LE	CC	MET+CC	FSH	hMG	BLOD
Abu Hashim,2011a	CC	hMG	FSH	MET+CC	LE	BLOD
Abu Hashim,2011b	MET+CC	FSH	hMG	LE	BLOD	/
Kaya,2004	CC	MET+CC	FSH	hMG	LE	BLOD
Liu,2015	CC	hMG	FSH	MET+CC	BLOD	LE
McFaul,1990	FSH	MET+CC	CC	LE	hMG	BLOD
Mehrabian，2012	hMG	CC	FSH	MET+CC	LE	BLOD

CC: clomiphene citrate, MET: metformin, LE: letrozole, FSH: follicle-stimulating hormone, hMG: human menopausal gonadotropin, MET+CC: metformin combined with clomiphene citrate, MET+LE: metformin combined with letrozole, ULOD: unilateral laparoscopic ovarian drilling and BLOD: bilateral laparoscopic ovarian drilling.
